# A Human-Centered Design Methodology to Enhance the Usability, Human Factors, and User Experience of Connected Health Systems: A Three-Phase Methodology

**DOI:** 10.2196/humanfactors.5443

**Published:** 2017-03-16

**Authors:** Richard Harte, Liam Glynn, Alejandro Rodríguez-Molinero, Paul MA Baker, Thomas Scharf, Leo R Quinlan, Gearóid ÓLaighin

**Affiliations:** ^1^ Electrical & Electronic Engineering School of Engineering & Informatics National University of Ireland Galway Galway Ireland; ^2^ HUMAN MOVEMENT LABORATORY CÚRAM SFI Centre for Research in Medical Devices NUI Galway Galway Ireland; ^3^ General Practice School of Medicine National University of Ireland Galway Galway Ireland; ^4^ Georgia Institute of Technology Center for Advanced Communications Policy (CACP) Atlanta, GA United States; ^5^ Irish Centre for Social Gerontology National University of Ireland Galway Galway Ireland; ^6^ Physiology School of Medicine NUI Galway Galway Ireland

**Keywords:** human-centered design, user-centered design, usability testing, user interface design, connected health, human factors, mHealth

## Abstract

**Background:**

Design processes such as human-centered design, which involve the end user throughout the product development and testing process, can be crucial in ensuring that the product meets the needs and capabilities of the user, particularly in terms of safety and user experience. The structured and iterative nature of human-centered design can often present a challenge when design teams are faced with the necessary, rapid, product development life cycles associated with the competitive connected health industry.

**Objective:**

We wanted to derive a structured methodology that followed the principles of human-centered design that would allow designers and developers to ensure that the needs of the user are taken into account throughout the design process, while maintaining a rapid pace of development. In this paper, we present the methodology and its rationale before outlining how it was applied to assess and enhance the usability, human factors, and user experience of a connected health system known as the Wireless Insole for Independent and Safe Elderly Living (WIISEL) system, a system designed to continuously assess fall risk by measuring gait and balance parameters associated with fall risk.

**Methods:**

We derived a three-phase methodology. In Phase 1 we emphasized the construction of a use case document. This document can be used to detail the context of use of the system by utilizing storyboarding, paper prototypes, and mock-ups in conjunction with user interviews to gather insightful user feedback on different proposed concepts. In Phase 2 we emphasized the use of expert usability inspections such as heuristic evaluations and cognitive walkthroughs with small multidisciplinary groups to review the prototypes born out of the Phase 1 feedback. Finally, in Phase 3 we emphasized classical user testing with target end users, using various metrics to measure the user experience and improve the final prototypes.

**Results:**

We report a successful implementation of the methodology for the design and development of a system for detecting and predicting falls in older adults. We describe in detail what testing and evaluation activities we carried out to effectively test the system and overcome usability and human factors problems.

**Conclusions:**

We feel this methodology can be applied to a wide variety of connected health devices and systems. We consider this a methodology that can be scaled to different-sized projects accordingly.

## Introduction

### Background

*Connected health* is a term used to encompass health care concepts such as eHealth, telehealth, telemedicine, and mHealth, and refers to the use of health technology to deliver health care to patients remotely [1]. Connected health products include blood pressure and heart rate monitors; diabetes management devices; thermometers; weighing scales; and, increasingly, fitness, diet, and activity trackers. All of these are characteristic of the *quantified-self* movement, a modern trend whereby individuals seek to track their own physical, behavioral, or environmental information [2]. These devices, systems, and services, when combined with an appropriate clinical-based information and communications technology infrastructure, can allow users to take control of their own health and wellness in their homes while maintaining contact with a health care professional. This model can do the following: support continuous health monitoring for both individuals and for whole groups; encourage healthy behaviors to prevent or reduce health problems; support chronic disease self-management; reduce the number of health care visits; and provide personalized, localized, and on-demand interventions [3].

An increasing focus on reducing health care costs for patients of all ages has spurred the growth of the connected health care market. In a Geisenger Health Plan study, it was found that postdischarge use of connected health monitoring for heart patients reduced readmission to hospital by 44% [4], while a similar study by Agboola et al reported similar decreases in heart failure-related readmissions of 48% in the first 30 days postdischarge [5].

Many connected health devices share common features; they are typically compact, electronic modules that carry out at least one specific health care function. These devices generally have buttons, switches, touch or nontouch screens, speakers, and wearable clips or belts; in addition, they are generally designed to measure some aspect of a person’s health status [6]. Connected health devices, such as wearable heart rate or blood pressure monitors, can be synced to mobile phones with the mobile phone acting as a data storage, data transmission, and interaction platform.

Connected health devices have various characteristics that make them unique compared to other health or medical devices that may be utilized in hospital, clinical, or surgical settings [7]. Connected health devices are designed to be used in an unsupervised manner in the home by users who may not be specialists in health care. Connected health devices have user interfaces (UIs) that require some level of human-computer interaction and they comprise software and hardware elements. Due to the likely use of these devices by disabled, elderly, or infirm users, connected health devices require UIs with good usability characteristics. There may be different levels of interaction required, in terms of both complexity and regularity, across a range of devices.

The UI features of connected health devices can place demands on users that are incongruent with their capabilities [6]. It has been observed that many otherwise excellent products have failed in the marketplace due to poor interface design, while mediocre products have flourished due to attractive and user-friendly interface design [8]. Therefore, an important consideration in the design of connected health devices is the usability and human factors characteristics of the device interfaces and, hence, the user experience they provide for the user.

Usability is defined by the International Organization for Standardization (ISO) as “the extent to which a user can use a product to achieve specific goals with effectiveness, efficiency and satisfaction in a specified context” [9]. The term *human factors* is described by the American National Standards Institute and the Association for the Advancement of Medical Instrumentation as “the application of knowledge about human capabilities (physical, sensory, emotional, and intellectual) and limitations to the design and development of tools, devices, systems, environments and organizations” [10]. User experience is defined as the perceptions and responses of users that result from their experience of using a product or service [11]. Both the Food and Drug Administration (FDA) and the Agency for Healthcare Research and Quality have called for usability and human factors evaluation of medical devices and systems during the design process [12,13], with the FDA requiring evidence of end user involvement during the design process when reviewing market presubmissions [14].

### User- and Human-Centered Design

**Figure 1:**
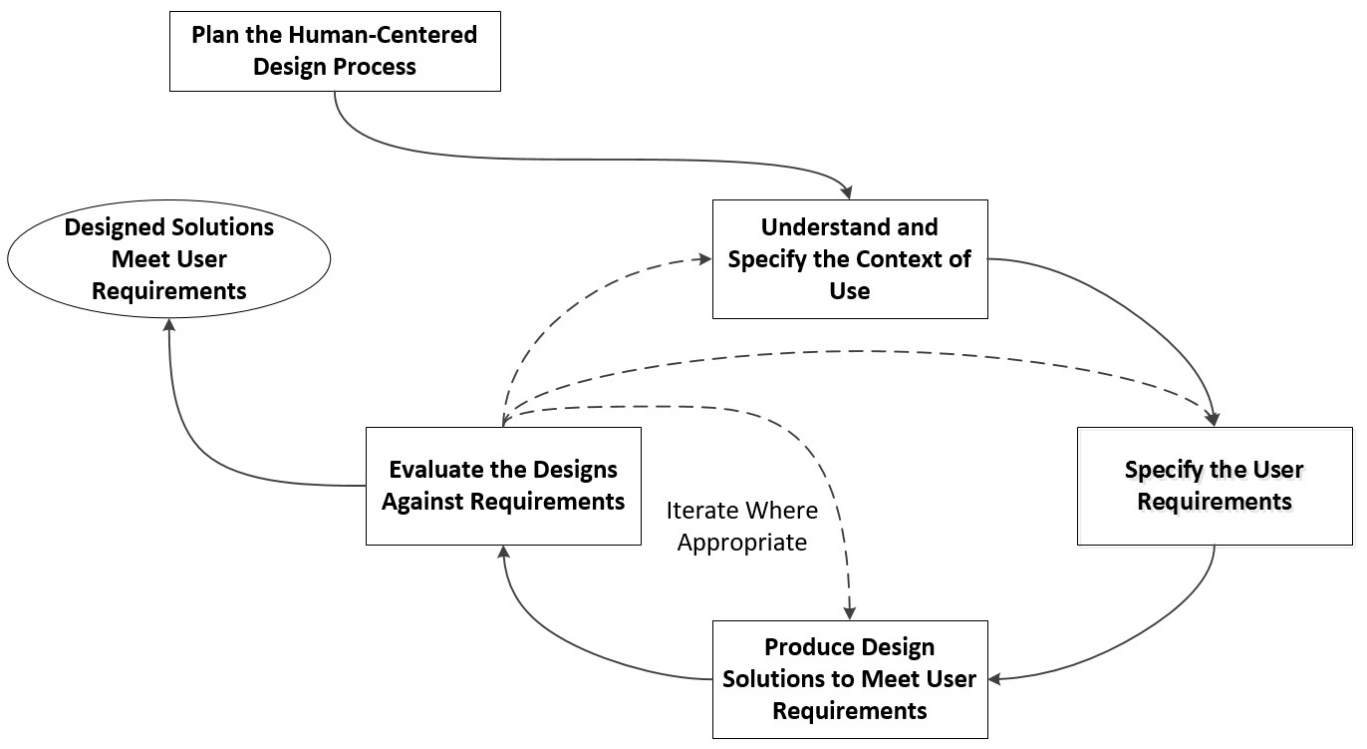
Human-centered design has four main activity phases: (1) Specify the user and the context of use; (2) Specify the user requirements; (3) Produce design solutions; and (4) Evaluate designs against requirements.

User-centered design (UCD) is a design philosophy that seeks to place the end user at the center of the design process. The term was coined in the 1980s by Donald Norman [15] who put forward guidelines that designers could follow in order for their interfaces to achieve good usability outcomes. From that point on, many designers, researchers, and policy makers have proposed various methodologies and techniques that seek to involve the end user in the design process, with the end user being defined as the “person who will ultimately be using the product.” In their 2010 standard ISO 9241-210, the ISO extended the definition of UCD to “address impacts on a number of stakeholders, not just those typically considered as users,” referring to the design approach as human-centered design (HCD) [11]. As such, we will refer to UCD as HCD from now on in this paper. The ISO 9241-210 standard defines human-centered design as “an approach to systems design and development that aims to make interactive systems more usable by focusing on the use of the system and applying human factors/ergonomics and usability knowledge and techniques.” The standard also describes the potential benefit of following a design approach that improves usability and human factors: “Usable systems can provide a number of benefits, including improved productivity, enhanced user well-being, avoidance of stress, increased accessibility and reduced risk of harm.” Putting the user at the core of the design process is also the guiding principle of a philosophy related to HCD, that of *universal design*. The aim of universal design is to create accessible products, environments, and services for all users regardless of their physical or cognitive abilities [16]. It must be noted that this goal is not always the main goal of HCD, which may try to make a product accessible to a specific target group of end users, rather than all user groups [17]. HCD has four defined activity phases: (1) Identify the user and specify the context of use; (2) Specify the user requirements; (3) Produce design solutions; and (4) Evaluate design solutions against requirements. HCD has roots in the field of *requirements engineering* in that it seeks to document the user requirements and how they are being met by the design at each stage of development [18,19]. The main goal of HCD is to increase the usability of the product in order to create maximum user satisfaction and increase the safety performance of the device. The process model of HCD as defined in ISO 9241-210 is illustrated in Figure 1.

As well as the steps outlined above, there are six requirements which are described in ISO 9241-210 that a process must meet if it is to be considered an HCD process. Our methodology before meeting any other requirements must meet these six requirements. We will refer to these requirements as Requirements 1-6, which are as follows: (1) The design is based upon an explicit understanding of users, tasks, and environments; (2) Users are involved throughout design and development; (3) The design is driven and refined by user-centered evaluation; (4) The process is iterative; (5) The design addresses the whole user experience; (6) The design team includes multidisciplinary skills and perspectives.

### Human-Centered Design for Connected Health Devices

So far, we have discussed the increasingly important role of connected health devices in health care globally [20]. We have established that various connected health devices have interface characteristics that could cause problems for older adult users or users with disabilities [6]. We have also established that as medical devices, connected health devices and systems are unique in terms of context of use and UI requirements [7]. Finally, we have outlined the technical aspects and requirements of HCD. This leads us to the question, “Why is all of this important for connected health system design?” In the context of what has just been discussed, we think there is a need for a customized HCD methodology for the design of connected health devices; we will now further explore why we think this is necessary by highlighting three specific needs.

### The Need for Descriptive Detail and Standardized Structure for Human-Centered Design Methodologies Within Medical Literature

We must make it clear that various HCD approaches to the design of health care technology have been described in the literature. For example, Vermeulen et al described a multiphase HCD methodology for the design of an older-adult activity monitor, with the phases including the following: analysis of users and their context, identification of user requirements, development of the interface, and evaluation of the interface in the lab [21]. Schaeffer et al employed an HCD methodology where they used surveys and focus groups to gather user requirements and create interface prototypes for an insulin pump [22]. Castilla et al described an HCD process for a telepsychology app, where they presented end users with icon and interface concepts in the first step of their design process, before moving on to a cognitive walkthrough methodology to evaluate the navigation of the interface. These and many other examples like them [23-25] show the wide variance in the application of HCD to health devices and systems. It also exhibits the broad range of usability and human factors testing activities available to engineers and designers to gather feedback. Many of these activities are not new; many of the most well known testing and evaluation techniques had been developed by the late 1980s [26-29]. However, we feel that in a lot of the connected health literature, there is a lack of descriptive detail of the activities carried out within the design process, particularly in regard to ISO 9241-210, and a lack of reporting on how successful or unsuccessful these activities were.

### The Need for a Methodology That Allows for Rapid Development Cycles

Additionally, the connected health industry is seen as a fast-moving, highly competitive industry [30], highlighting a need not only for devices that achieve adequate levels of usability, but also for devices that can have rapid development cycles associated with them. This need is punctuated by the association of connected health technology with mobile devices, such as mobile phones. The phones themselves typically act as collection, transmission, and storage platforms for the health data, while the mobile phone apps provide users with an interface to their data or to an external device. In 2015, over 100,000 mobile health apps were available for download between the Google Play Store and the Apple App Store [31]. By 2016, over 500 million people are expected to be utilizing mobile health apps to some degree [32]. This proliferation of mobile health devices and apps means that these devices and their apps can become relatively obsolete in a short period of time [33], with a consequent need for shorter and shorter product lifecycles as was previously experienced in the software industry. This can mean that companies may not be able to incorporate a full HCD methodology into their product development cycle. In light of this observation, it is the authors’ opinion that presenting a detailed, comprehensive description of an HCD methodology is warranted, one that is in line with ISO 9241-210 and is optimized for use with connected health devices through the streamlining of the different steps in the HCD process.

### The Need for a Guided Approach That Emphasizes Planning and Documentation

It has been previously observed that developers of connected health solutions are in many cases more engaged with the technical innovation in these systems rather than with their usability [7,34]. More recently, it was identified that there is a need for guidelines on how to conduct the design and development process for connected health devices in terms of usability [35]. Finally, in the development of medical devices, appropriate documentation of the design process is critical, particularly if the device is to adhere to a standard such as that from the International Electrotechnical Commission (IEC), IEC 62366-1. The FDA requires evidence of end user involvement during the design process when reviewing market presubmissions [14].

Therefore, as well ensuring our methodology adheres to the six guiding principles of HCD as outlined in ISO 9241-210, we will add three more requirements that our methodology must meet. We will refer to these three new requirements as Requirements 7-9, and they are listed below:

1. Requirement 7: Our methodology will follow the steps outlined by ISO 9241-210 as closely as possible and give a detailed description of activities carried out and outcomes achieved in each phase.

2. Requirement 8: Our methodology will utilize activities that allow for rapid prototyping, testing, and development.

3. Requirement 9: Our methodology will emphasize planning activities in advance and generating the appropriate documentation.

In this paper, we will describe a three-phase methodology which follows the same process as outlined in ISO 9241-210 (see Figure 1), which adheres to the six requirements it outlines as well as the three additional requirements we have just derived. In the following section, we will provide a detailed description of our activities and the justification for them. We will also provide an example of the application of the methodology to a connected health system. This paper will not provide the results of this application as those results will appear in a related publication.

## Methods

### Overview

The methodology, which will be described in this section, now has nine requirements that must be fulfilled. These are listed below with appropriate elaboration:

1. Requirement 1: The design is based upon an explicit understanding of users, tasks, and environments. In the first phase of our methodology, we will establish context of use, user requirements, and user profiles.

2. Requirement 2: Users are involved throughout the design and development. We will involve end users and expert users as much as possible in each phase.

3. Requirement 3: The design is driven and refined by user-centered evaluation. We will use evaluation techniques at each phase to achieve measurable results.

4. Requirement 4: The process is iterative. We will have multiple phases where design changes can be made after each phase; the process can revert back to a previous phase if necessary.

5. Requirement 5: The design addresses the whole user experience. Use cases developed in the first phase will address all aspects of use and will be used as reference points before and after each phase.

6. Requirement 6: The design team includes multidisciplinary skills and perspectives. We will incorporate multiple perspectives from disciplines within the design team, from stakeholders, and from experts. Here we define stakeholders as any person involved in the project who is affected by the activities or outcomes related to the product in question. An expert is defined as any person with an expert knowledge of the product, the end user, or of usability and human factors.

7. Requirement 7: Follow the steps outlined in ISO 9241-210 and provide details of suggested activities and their expected outcomes within each phase. Our phases will be structured to conform to the phases outlined in ISO 9241-210 and will outline which activities should be carried out in each phase.

8. Requirement 8: Perform rapid development and testing while maintaining clear structure. The early phases of our methodology will designate activities that allow for rapid prototyping and evaluation.

9. Requirement 9: Our methodology will be well planned with all activities, outcomes, and design changes properly documented. Our methodology will seek to embed the documentation of all activities, design, and developments.

Based on these requirements, we will now describe a three-phase methodology that will fulfill these requirements. These three phases are labeled as follows:

1. Phase 1: Establishing Context of Use and User Requirements

2. Phase 2: Expert Inspections and Walkthroughs

3. Phase 3: Usability Testing With End Users

**Figure 2:**
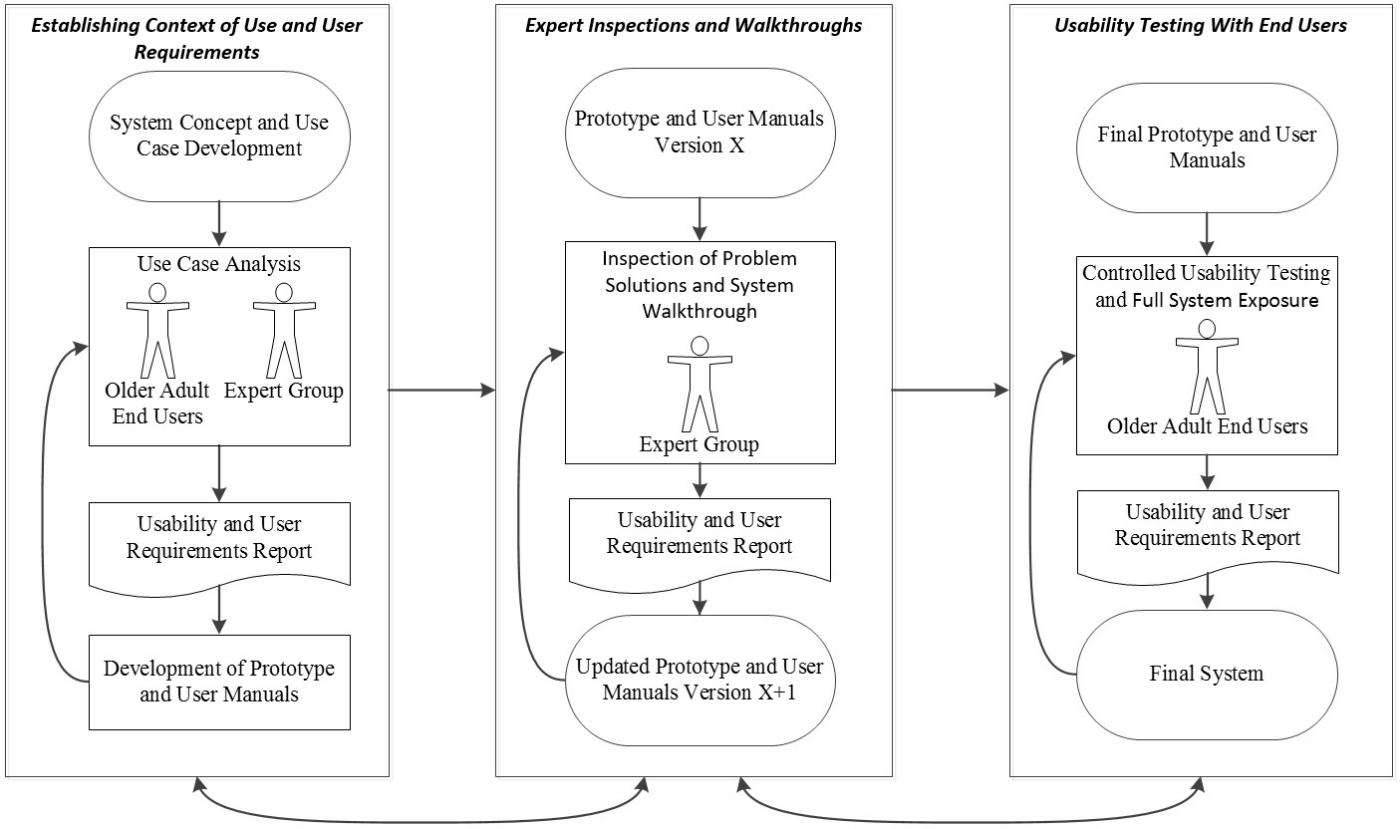
Our human-centered design approach to a connected health app.

The full methodology is illustrated in Figure 2 and then described in further detail within the text.

### Phase 1: Establish Context of Use and User Requirements

#### Overview

This phase establishes the context of use of the device and the requirements and needs of the target end user. Usually in early phase testing, to understand the needs of the user, activities such as interviews [36], surveys, and ethnographic observations are carried out [37,38]. This can be resource intensive and difficult to document properly. In our methodology, we attempt to gain an explicit understanding of users, tasks, and environments (Requirement 1) through the immediate construction of a use case document. Constructing use cases is a commonly used method to analyze user requirements and user preferences [39,40,23]. Starting with the system concept as reference point, the use case document should be made up of flow diagrams, storyboards, screenshots, interface mock-ups, paper prototypes, and descriptive end user profiles. The document is designed to be interactive and descriptive; it is designed to provide a common platform for project stakeholders to communicate their vision for each component’s and user’s role within the system and the interactions they have with each other, thereby attempting to address the whole user experience (Requirement 5). User profiles should be drawn up within the use case document of potential users, describing capabilities, requirements, and preferences.

#### Suggested Activities

These use cases can be exposed to a group of experts with knowledge of the system and/or usability (Requirement 6) and to a group of end user representatives (Requirement 2) [41]. At various points in the document, questions can be put to the reader or they can share their insights; in this way, the use case analysis acts like an interview, survey, and ethnographic exercise all in one, allowing for more rapid turnaround of information related to user requirements (Requirement 8). In the early phase of the design process, designers could pursue many different possible solutions and concepts. Within the use case, or as an accompaniment to it, paper prototypes, wireframes (essentially a skeletal framework of an interface, usually a website), and mock-ups should be exposed to the users [42-45]. Likert scales can be used to query the reader’s agreement with aspects of the prototypes (Requirement 3).

#### Outcomes

A usability report and a list of user requirements, backed up by quantitative and qualitative data, are produced (Requirements 3 and 9). Semifunctioning prototypes or mock-ups that fulfill as many of the uncovered requirements as possible should now be built and made available for testing in Phase 2. The first user manuals, if required, should also be ready for inspection in Phase 2. This phase fulfils Requirements 1-3 and 5-9.

### Phase 2: Expert Inspections and Walkthroughs

#### Overview

The testable prototype should now be exposed to a controlled formative test that takes into account usability, human factors, and overall user experience characteristics, as well as testing the overall functionality of the prototype (Requirements 3, 5, and 6). This can be done using so-called discount usability techniques to ease the burden on time resources and to forgo expensive recruitment of end users (Requirement 8). The testing is carried out with reference to the use case and the requirements generated from Phase 1. Problems uncovered by the tests need to be prioritized and addressed in turn by the development team, with testing repeated if necessary (Requirement 4).

#### Suggested Activities

Evaluation and inspection methods could be carried out. Usability inspection involves a multidisciplinary expert group (Requirement 6) inspecting the interface and attempting to identify usability and human factors problems [23]. This can be in the form of a heuristic evaluation where the interface is compared to a set of predefined design guidelines [45,46] or a cognitive walkthrough [47,48]. In a cognitive walkthrough, the expert group can carry out a task by way of task analysis of the interface while focusing on cognitive processes that the task requires, documenting where they encounter problems. Usability inspections are commonly used as a precursor to formal end user testing [49-51] because they are seen as low cost and easily implementable techniques than can garner quick and concise feedback [52]. Their flexibility and quick feedback lend themselves well to the evaluation of almost any type of system or device. In addition, usability inspections have been used to assess the usability of electronic health record systems [53], Web-based interfaces for telemedicine apps [54], online educational websites [55], infusion pumps [56], pacemaker programmers [57], instrumented insoles [51], and mobile phone apps [58].

#### Outcomes

An updated usability report is produced (Requirement 9). A now-advanced prototype with almost full functionality with accompanying user manuals should now be ready for testing with end users. This phase fulfils Requirements 1 and 3-9.

### Phase 3: Usability Testing With End Users

#### Overview

The now-advanced prototypes are exposed to end users in summative user testing (Requirement 2). The test can be carried out in controlled settings like a lab, but it is more useful to carry out field-testing with end users, such as in their homes. Problems uncovered by the tests need to be prioritized and addressed in turn by the development team, with testing repeated if necessary (Requirement 4). Test cycles should be kept short with a low number of participants in each cycle if possible.

#### Suggested Activities

User testing should be carried out; it has been greatly described in the literature [59-61] and involves monitoring users while they interact with the system interface. This monitoring can be carried out in different environments, with laboratory sessions allowing for more control over the experiment and more robust data, albeit with the loss of real-world fidelity. Observing users in a more natural use environment can lead to richer data, but the data can be harder to quantify effectively. In early instances of user testing, the administrator will often ask the subject to think aloud, allowing the observer to gain an insight into the train of thought the user is employing as they encounter and attempt to overcome usability and human factors problems [62,63] (Requirements 1 and 5). Cameras, audio recorders, and note taking are employed to record the user behavior. Scales such as the Quebec User Evaluation of Satisfaction with Assistive Technology, the System Usability Scale (SUS), the After-Scenario Questionnaire (ASQ), the NASA Task Load Index (TLX), and the Visual Analogue Scale, as well as 5-point Likert scale questionnaires [64], are utilized to record and quantify user satisfaction (Requirement 3). An example of a Likert questionnaire item might be “I can read the text on the screen without any difficulty”; a user will then rate their level of agreement or disagreement with the item on a scale of 1-5. Efficiency and effectiveness are measured by recording time taken to complete tasks and error and completion rates [65].

#### Outcomes

A very advanced prototype that can be subjected to further user testing or expert inspection can be carried out if required. This phase fulfils Requirements 1-5 and 7-9.

Within each phase, activities can and should be repeated if necessary (Requirement 4). After each phase, all problems are recorded and documented in structured usability and human factors reports, or another form of presentation, so that all stakeholders are aware of the problems and all problems and changes are documented (Requirement 9) [66].

### Application of Methodology to a Connected Health System

#### Overview

**Figure 3:**
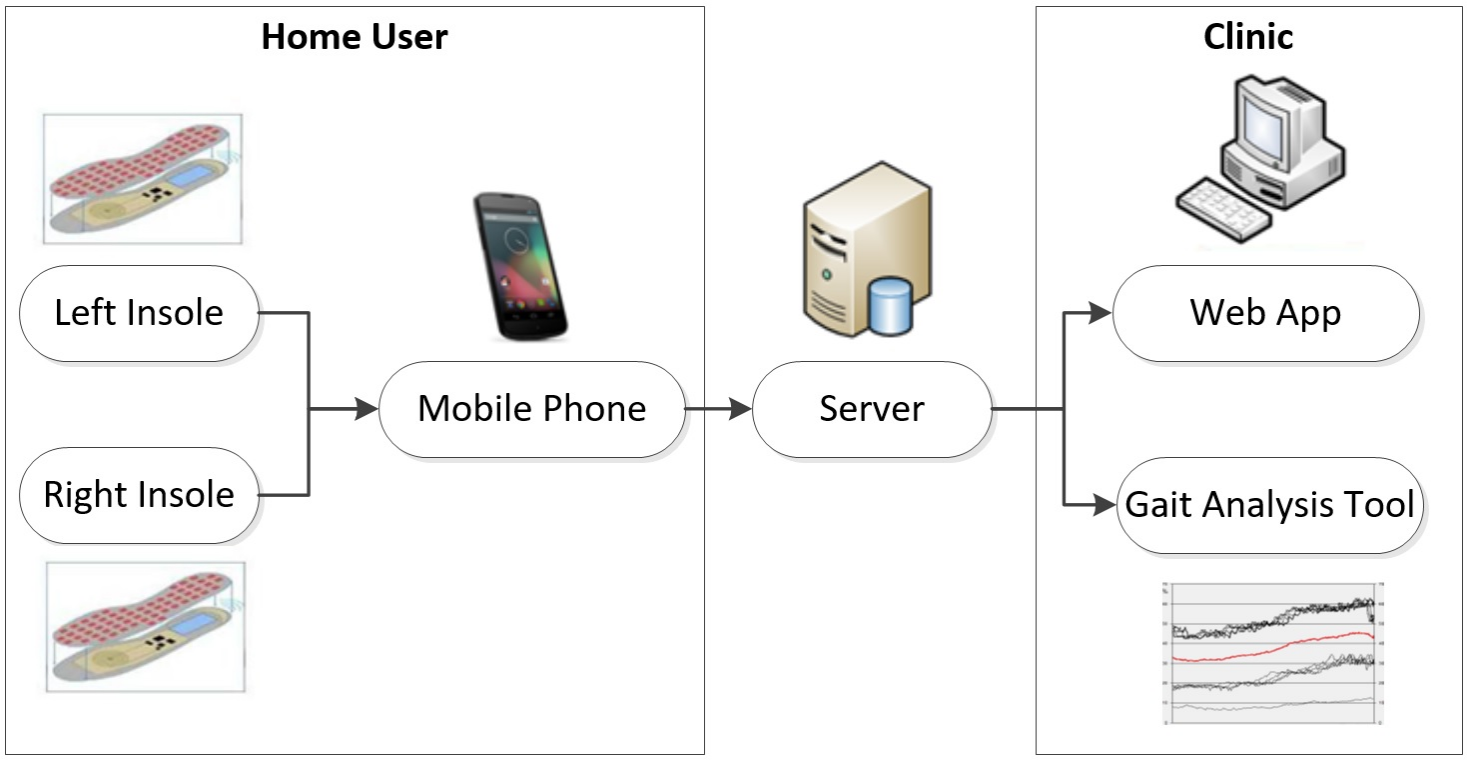
The Wireless Insole for Independent and Safe Elderly Living (WIISEL) system.

This methodology was applied to assess and enhance the usability, human factors, and user experience of a connected health system known as the Wireless Insole for Independent and Safe Elderly Living (WIISEL) system, a system designed to continuously assess fall risk by measuring gait and balance parameters associated with fall risk [67]. The system is also designed to detect falls. The architecture of the system is illustrated in Figure 3 and it is described in further detail below.

It is proposed that the system can be used in the home by a user for a period of time in order to identify specific gait and balance patterns that may be affecting a user’s fall risk. The system is targeted at older adults who represent a high-fall-risk group. The system consists of a pair of instrumented insoles worn by the user and a mobile phone carried by the user. Data collected by embedded sensors in the insoles are sent to the mobile phone, where they are then uploaded to a server in a clinic for processing and analysis. The mobile phone represents a major interface in the system, as this is how the home user will primarily interact with the WIISEL system with the WIISEL app allowing the user to check the system status, sync with the insoles, send data to their local clinic, and monitor their daily activity.

##### Phase 1 Activities

**Figure 4:**
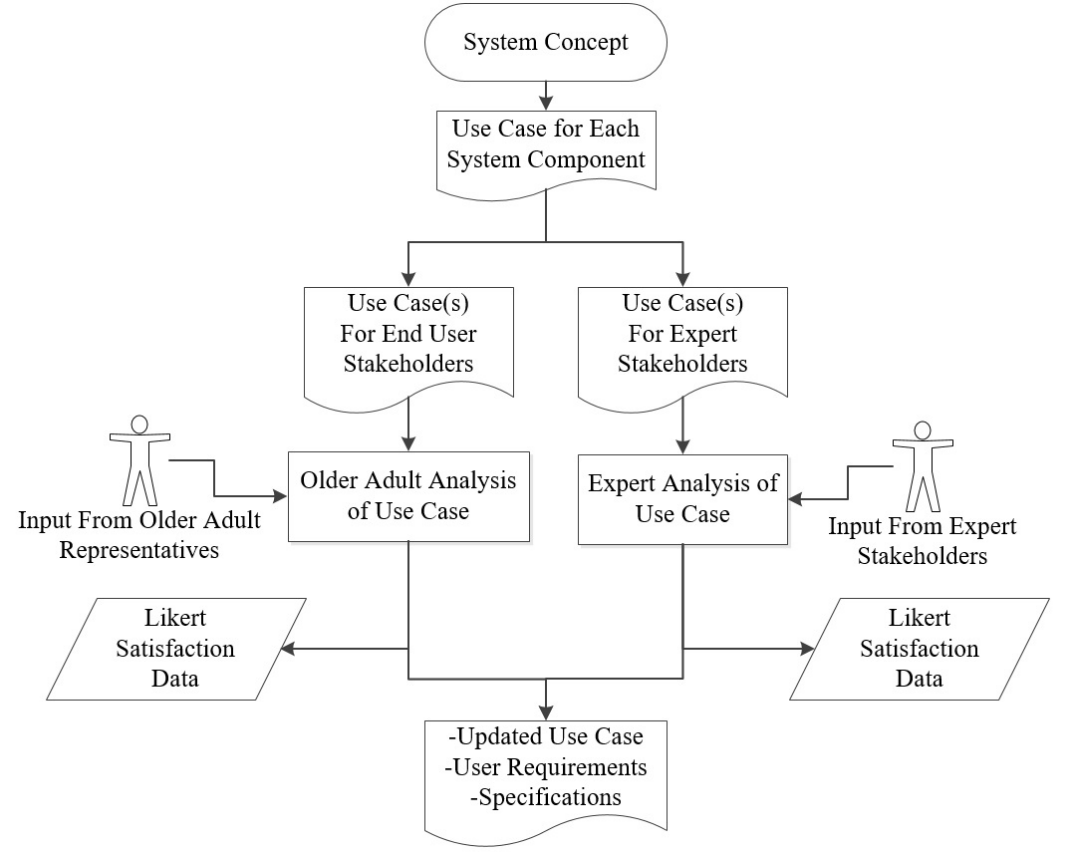
Phase 1 activity flow.

The process of Phase 1 is summarized and illustrated in Figure 4.

##### Use Case Creation

**Figure 5:**
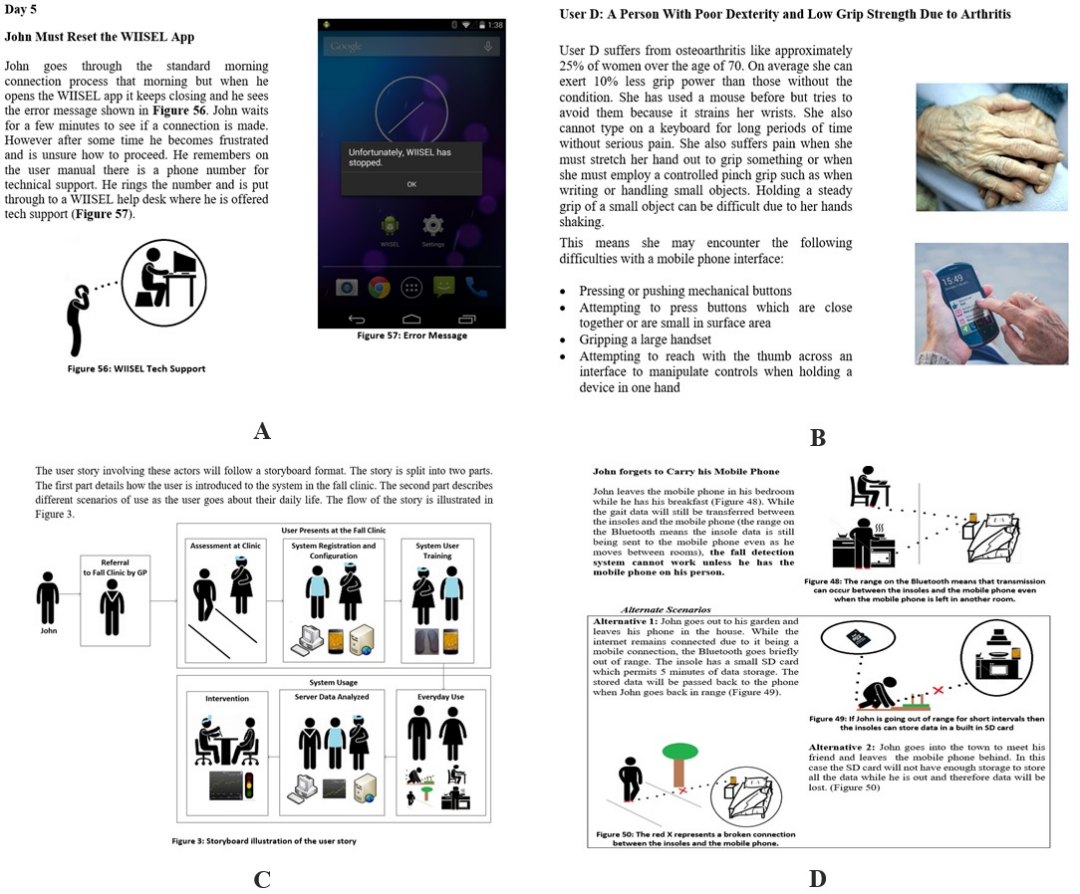
Examples of the information included in the WIISEL use case. (A) A scenario presented in the use case where the user, John, must carry out a troubleshooting sequence with the app; a life-size color screenshot of the mobile phone interface is shown. (B) A section of the use case that profiles typical physical capabilities of the target user and how this might affect their interaction with the mobile phone. (C) A storyboard at the beginning of the document summarizing the whole process, from when the user is prescribed the system to when they return to the clinic having worn it for a period of time. (D) A scenario in the use case where it describes what might happen to the phone while the user is doing daily home chores. WIISEL: Wireless Insole for Independent and Safe Elderly Living; GP: general practitioner.

The use case document was constructed with inputs from all project stakeholders, who were able to share their opinions on how the system would work and what it would be used for. Scenarios were described in the document, which identified the tasks the user must carry out with the system, the order the tasks were carried out, and the context in which the tasks were carried out. Potential risks that the user might encounter through their interaction with the system were also identified (using ISO 62366 as a reference guide). Examples of the information included in the WIISEL use case are illustrated in Figure 5.

##### Use Case Analysis

**Figure 6:**
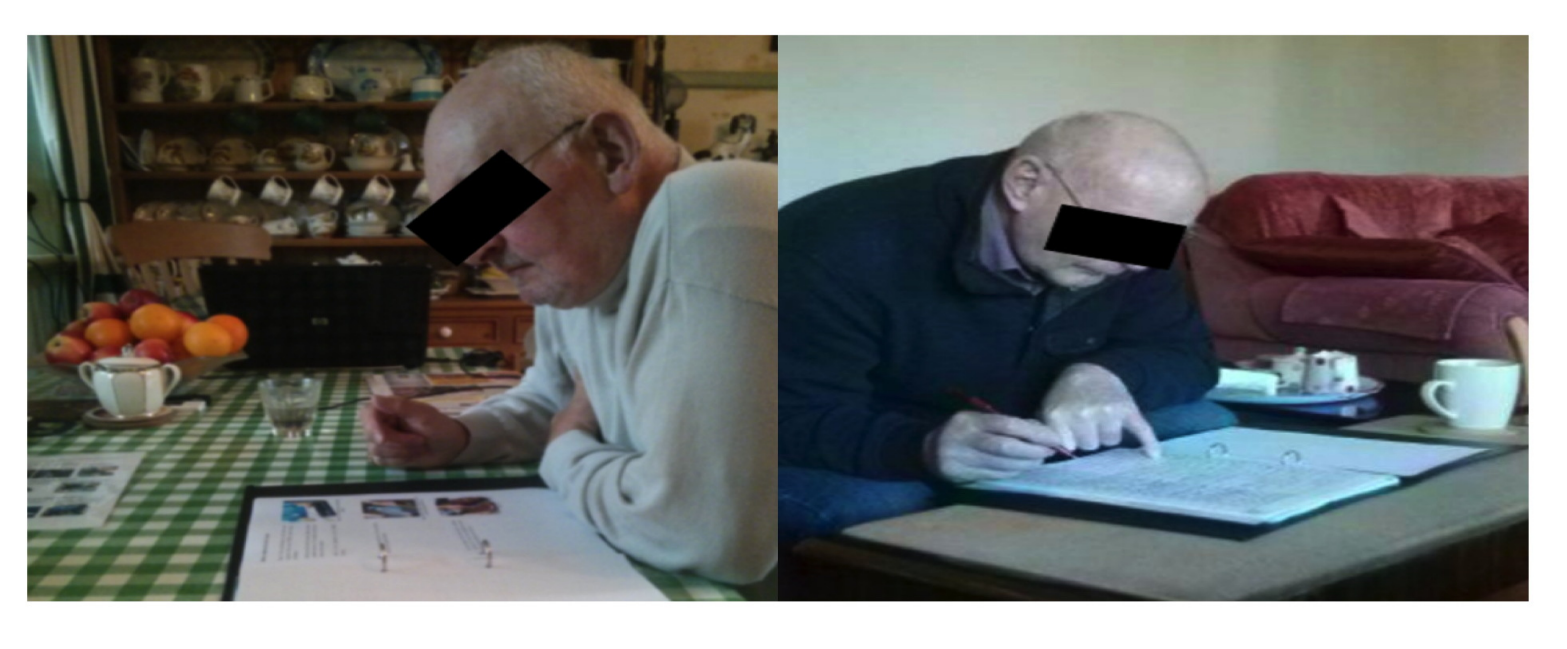
Older adult participants analyzing and providing feedback on the use cases.

The use cases were examined by a series of stakeholders, which included target end users—older adults and health professionals—and people with relevant expertise who may not necessarily be end users but who have experience in the design of similar systems. The reader examined the scenarios one after another. After each scenario of the use case, the reader was interrogated on their thoughts on what they had seen using tick-box Likert scales which were embedded in the document. For example, in the case of the use case describing the use of the WIISEL mobile phone, the user filled out Likert scales that queried their opinions on color schemes, text size, button size, and screen navigation flows as observed from high-definition color screenshots. Examples of end users interrogating use cases and filling out the appropriate scales are shown in Figure 6.

Apart from the set scales the reader filled out, the think-aloud protocol was also employed by the reader so that they could elaborate on any potential problems and digress if necessary to related problems not explicitly presented in the use case.

##### Problem Classification

There are a number of methods to classify usability problems [68-70]. Many of these methods, such as clustering, heuristic evaluation, and Nielsen’s classifications, prove effective in identifying how likely an identified problem is to affect the user’s interaction with the system. Because the use case is not representative of the fully interactive system, it is not possible to carry out a traditional classification by observation and evaluation; rather, we used the transcripts and the scoring from the Likert scales to predict potential problems. A three-step process was employed:

1. *Clustering Identified Problems*: Using the compiled transcripts from the think-aloud protocol, we grouped explicit identification of problems on a scenario-by-scenario basis. Problems can be grouped according to a set of heuristics, making the problems easier to classify and track throughout the design cycle. In the case of the WIISEL mobile phone use case, the following set of heuristics (a-e) was used [70]:

(a) *Consistency/Clarity of Task Structure*: The flow of the task or the interface may cause confusion or may be hard for a typical user to follow.

(b) *Completeness and Sufficiency of Task Meaning*: Feedback obtained when the user carried out an action, or was required to carry out an action, was unclear or may cause confusion.

(c) *Noticeability*: An element on the interface that is important to the completion of the task is difficult to notice.

(d) *Discernibility*: Physical interface characteristics such as text size, button size, and color scheme—each of which is a subcategory—may make it difficult for the user to complete the task.

(e) *Cognitive Directness*: The user was required to carry out an action that did not result in the expected outcome.

2. *Relate Problem to Likert Item*: The identified problems were related to one of the Likert items put to the participants at the end of each use case scenario. The Likert items are related to each of the categories above.

3. *Calculate Severity Rating*: The median score was calculated for the Likert item (adjusted range 0-4, with 0 considered a perfect score and 4 considered the most severe). This provided a problem rating for the problem.

**Figure 7:**
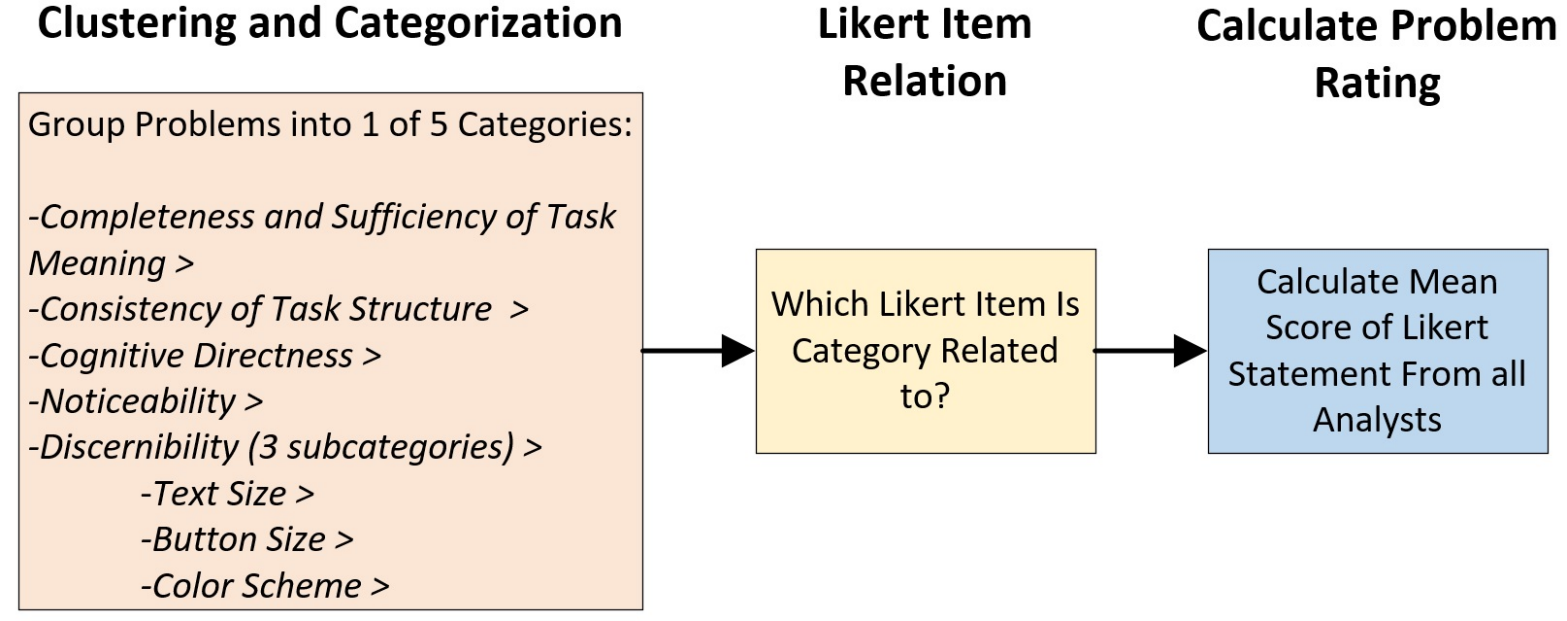
Structured process for prioritizing usability problems.

The methodology, illustrated in Figure 7, is sometimes referred to as bottom-up clustering because it groups together similar problem descriptions from first principles.

This list of problems can be dealt with straight away, as most of them will be aesthetic and superficial, while more complex problems, such as ones related to concepts and flow, can be further explored in functioning prototypes.

###### Phase 2 Activities

**Figure 8:**
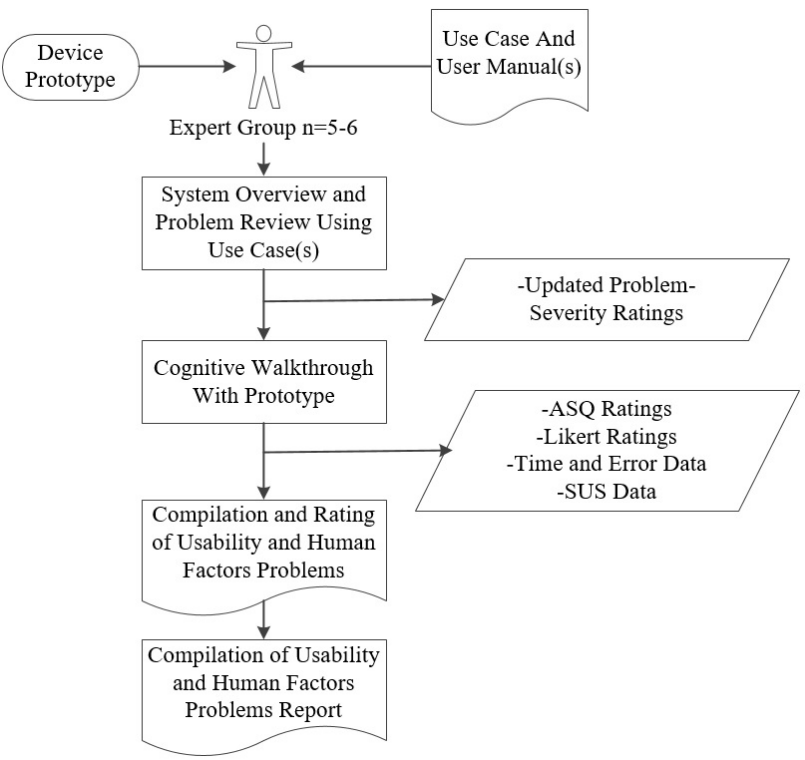
Phase 2 activity flow. ASQ: After-Scenario Questionnaire; SUS: System Usability Scale.

The Phase 2 activity flow is illustrated in Figure 8.

##### Inspection of Updated Use Case

In response to the feedback from Phase 1, a semifunctioning WIISEL mobile phone app prototype was also developed with accompanying user manuals—Working Prototype Version 1—and made available for expert walkthrough. An updated use case was also created to accompany the inspection—Paper Prototype Version 2. The original experts from Phase 1 carried out a two-part usability inspection. First, the experts inspected the solutions to the problems they had identified in Phase 1 using the new version of the use case—Paper Prototype Version 2—as a guide. This use case only presented the problems that the experts identified in their original analysis and showed how the problems had been addressed. Second, they inspected the physical app—Working Prototype Version 1—utilizing a walkthrough methodology.

The use case inspection consisted of four steps:

1. The expert was presented with the original use case scenario—Paper Prototype Version 1—in which they originally identified the problem. This provided the problem context.

2. The expert was presented with a description of the problem they identified within the scenario with, where possible, an annotated screenshot of the interface outlining where exactly the problem was identified.

3. The updated interface—Paper Prototype Version 2—was presented to the expert, which has sought to address the problem.

4. The expert was asked to mark the relevant Likert item for the purpose of calculating a new problem rating.

The expert was notified before proceeding that they could still reject any changes to the interface as being either inadequate or not being what they had suggested. The new problem ratings calculated from the Likert items filled out in Step 4 were then compared to the original ratings.

##### Cognitive Walkthrough With Manuals

**Figure 9:**
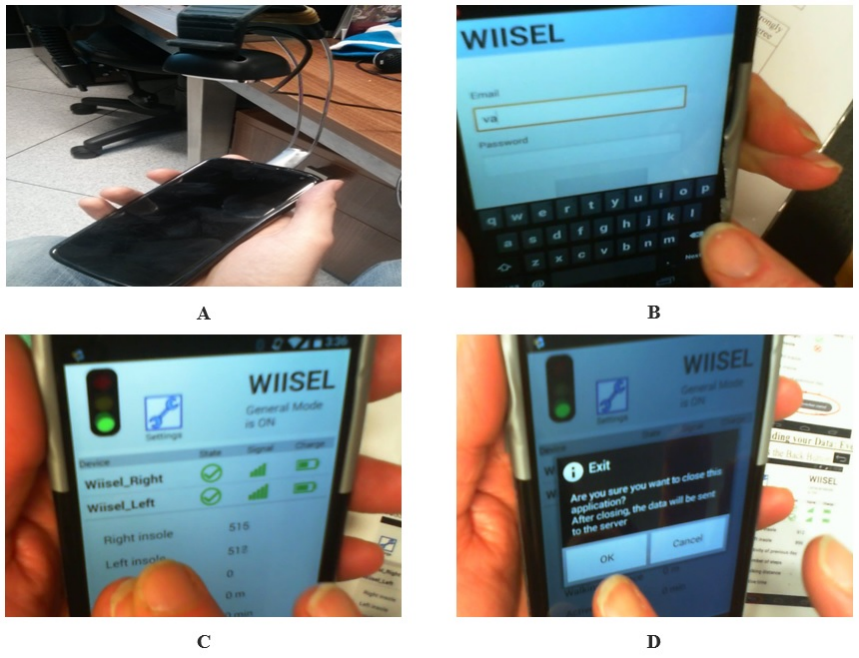
Phone screen interface. (A) The experts walk through each scenario in the user manuals with the phone; the cradle camera captures all of their interactions with the mobile phone. (B) An expert attempts to log in to the mobile phone app. (C) An expert follows the connection sequence from the user manual. (D) An expert carries out the data upload sequence.

In order to give the expert a chance to fully analyze the physical app and transition from a high-fidelity paper prototype to a functioning physical prototype, the app was presented to the expert following a cognitive walkthrough methodology. The cognitive walkthrough method is employed as a means of identifying usability problems in interactive systems, with a primary focus on determining how quickly and accurately new users would be able to complete a task with a system. A lightweight overhead camera (Microsoft Life HD+Mic) was attached using a wire cradle to the phone handset, which captured all interactions with the phone screen interface (see Figure 9).

The experts were walked through the user manuals and the app by the researcher as if they were a first-time user and were then asked to carry out a number of scenarios. They could consult the user manual at any time, but were not prompted by the administrator. They were encouraged to think aloud as they carried out each task. A number of usability metrics, such as time taken to complete task, errors made, and completion rate, were recorded during the walkthrough and captured using the overhead camera. The ASQ was employed after each scenario. The ASQ is a 7-point scale where a score of 7 indicates strong disagreement and 1 indicates strong agreement; a lower score indicates increased satisfaction with the interface. It seeks the user’s agreement on three statements related to key usability metrics: “Overall I am satisfied with the ease of completing this task,” “Overall I am satisfied with the amount of time it took to complete this task,” and “Overall I am satisfied with the support information (online help, messages, documentation) when completing this task.” All observed problems were again recorded and compiled in a usability and human factors report.

#### Phase 3 Activities: User Testing

**Figure 10:**
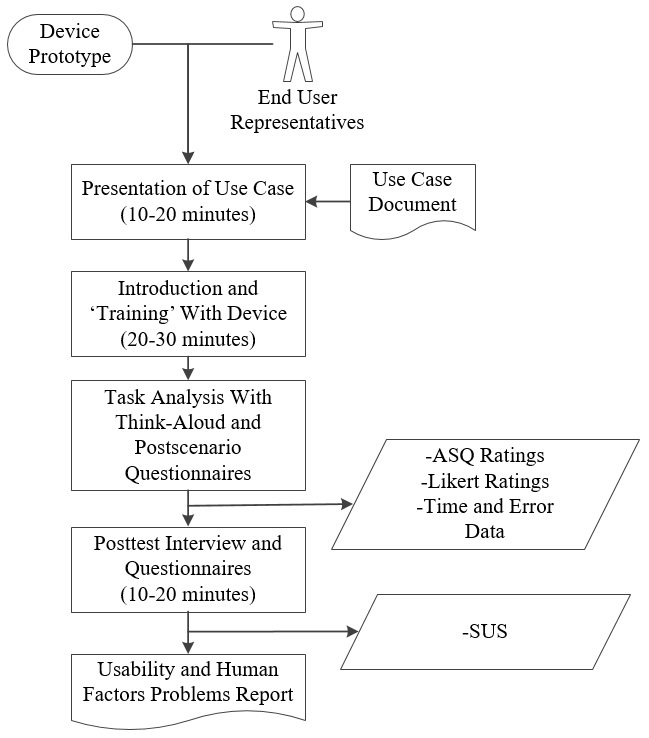
An example of Phase 3 activities. ASQ: After-Scenario Questionnaire; SUS: System Usability Scale.

**Figure 11:**
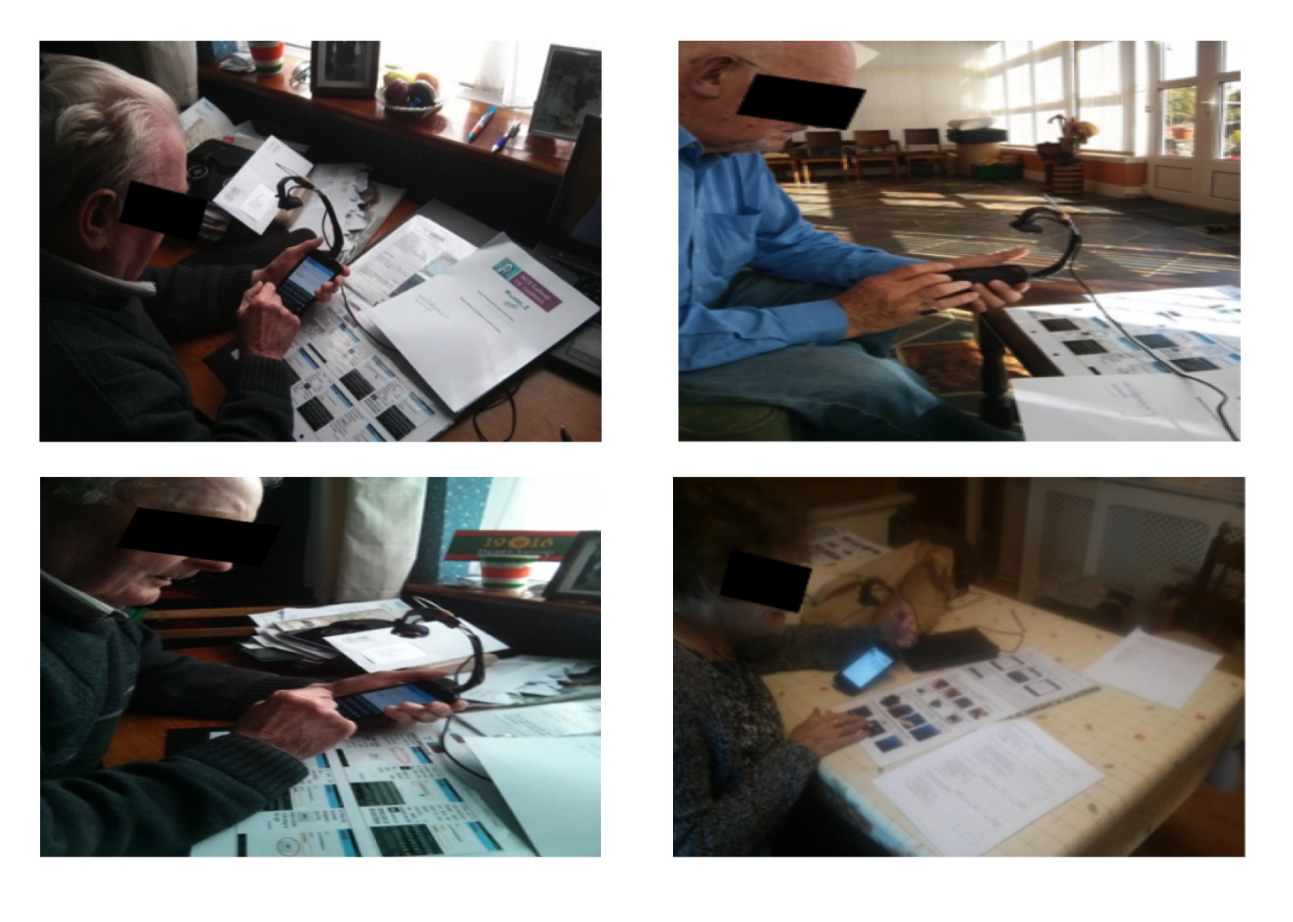
Older adult users carrying out tasks using the user manual as a guide during the user testing phase.

The process of Phase 3 is summarized and illustrated in Figure 10. In this phase, a now-advanced functioning prototype complete with user manuals where necessary was exposed to end users in controlled summative user testing. Any major problems with the system identified in the expert inspection should have been addressed by this time, particularly any problems that could adversely affect the health of the end user. The new manuals and updated interface—Working Prototype Version 2—were exposed to 10 older adults who had previously analyzed the use case. The testing was carried out in the home of the participant. The procedure was as follows:

1. The participant was asked to complete all tasks defined in the original use case.

2. Each task was carried out three times.

3. Before the testing began, the participants were guided through the task by the researcher using the user manuals. Allowing the participant to become familiar with the interface is important to separate genuine usability problems from mistakes due to unfamiliarity with the interface or device.

4. The overhead camera was attached and the screen interaction was recorded. No prompts were given to the participants, who were expected to complete the task using only the user manual as a guide (see Figure 11).

The same usability metrics were captured as in Phase 2 and the users were also interviewed posttest to get their general feelings on the device and interface. The feedback from user testing was used to generate the first working system complete with user manuals. Another usability report was compiled for the consumption of all stakeholders.

### Method Overview

**Figure 12:**
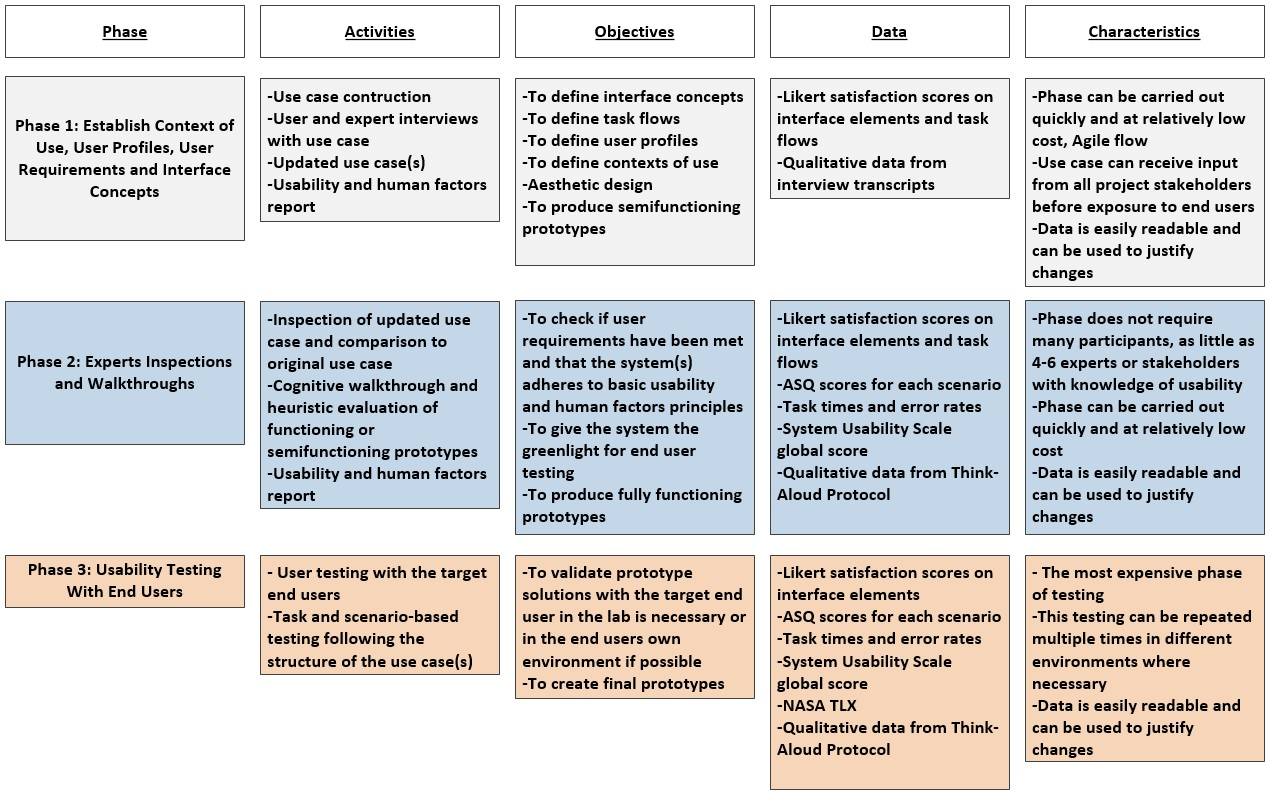
An overview of the complete methodology and all the suggested activities in each phase as applied to the Wireless Insole for Independent and Safe Elderly Living (WIISEL) system. ASQ: After-Scenario Questionnaire; TLX: Task Load Index.

The complete methodology, with a breakdown of each phase, is illustrated in Figure 12.

## Discussion

### Principal Findings

We have presented in detail the HCD methodology we consider to be a sensible and robust approach to designing interactive connected health devices. We will now review our proposed methodology and its example application to the WIISEL system by comparing the outcome to the nine requirements that were originally derived.

### Did Our Methodology Meet Our Requirements?

In terms of the first six requirements, we implemented a three-phase methodology that followed the flow of ISO 9241-210. The three phases allow for design iteration and can be repeated if necessary. The phases where iteration is most likely to occur are Phase 2 and Phase 3 [51], as these are the major testing phases with measurable outcomes, where outcome metrics can be compared when tests are repeated after prototypes have been updated. The methodology began with a phase that sought to gain an explicit understanding of users, tasks, and environments and tried to address the whole user experience by constructing a use case. This use case allowed for end users and multidisciplinary experts to become involved and evaluate the system concept, prototype screens, and the user task flow. The use case we developed for WIISEL contains information regarding the typical capabilities of the user, possible risks a user may encounter (eg, using ISO 62366 or ISO 14971 as a reference), what might happen if an error arises, and how often they would be expected to interact with the system. These aspects of system use were then explored in more detail in Phases 2 and 3, using the original use case as a reference point. The target end user was involved in Phase 1 and Phase 3. The end users in Phase 1 were able to provide accurate feedback on their user profiles and the context of use in which they would use the system, as well as provide early feedback on interface concepts and task flows. In Phase 3, we were able to closely observe them performing the system tasks that had been carefully designed in the previous two phases. In total, 22 end users were involved in our process. We successfully integrated multidisciplinary inputs into our design, utilizing experts from various backgrounds such as computer science, medicine, nursing, gerontology, psychology, and design. The experts should be chosen based on the type of system being designed and who the target end user is. In our case, the input of gerontologists and nurses with experience in technology for older adults was invaluable. If the necessary experts are not available, then generic inspectors should inspect the prototype using pre-established heuristics.

In terms of the three further requirements that were derived to add to the original six, ISO 9241-210 was used as a guiding source by following the principles and steps outlined within it to fulfill Requirement 7. To fulfill Requirement 9, before the process began we set out exactly what testing and design activities we were going to carry out. While there are many activities usability engineers can employ to test products, it is never necessary to try to use all of them in the same project. We felt it was best to choose what activities would best suit our particular device and project. It is important to plan and document the activities in a design file, particularly if the device is to adhere to a standard such as IEC 62366-1. Regular meetings were carried out among stakeholders and developers to discuss upcoming activities and design changes. After each activity, all results and findings were placed into presentable formats, such as PowerPoint slides, so they could be disseminated among team members and stakeholders. Methodologies for activities were also disseminated such that changes could be made before activities took place. To fulfill Requirement 8, in Phase 1 we carried out a well-planned and choreographed use case analysis activity that was designed to allow for rapid idea and concept exchange. The use case analysis acted like an interview, survey, and ethnographic exercise all in one because it was addressing the whole user experience and allowed end users, experts, and stakeholders to participate in the formation and analysis of concepts and ideas, as well as providing validation on user profiles and context of use. We utilized paper prototypes extensively in Phase 1 and usability inspections with small expert teams in Phase 2. This use of so-called discount usability engineering methods again allows for rapid turnaround times on prototypes and quick feedback to be sent to the design team. The use cases can be constructed in a matter of days, while a full use case analysis can be carried out with an end user or expert in an hour. The data are easy to process because all the data—the Likert data and think-aloud transcripts—are at hand from the one analysis and are relatable directly to the context of use.

### Final Comments and Limitations

We can say on a preliminary basis that all the objectives we originally outlined for this methodology have been successfully met. We feel that our proposed methodology, and the examples of its implementation in this paper, will provide prospective designers with a methodological blueprint to follow an HCD process that adheres to a standardized structure, but also allows for rapid development cycles.

We have also recognized some possible limitations in our methodology that need to be addressed. In Phase 2, we only tested the prototypes with experts from various disciplines. There are a number of reasons for this. First, as a matter of principle in terms of ergonomic quality control and safety, we feel it is important to not expose a prototype to a potentially vulnerable user group, such as older adults in this case, until it has been fully inspected and walked through by experts. The example of a mobile phone app may not seem necessary to warrant this level of caution; however, we want this methodology to be applicable to all kinds of connected health devices, some of which may have greater levels of risk than others. Second, the expert input in Phase 2 allowed for a fresh third-party perspective on the system and brought a level of expertise in areas of usability, human factors, and interface design, something that the target end user themselves may not have experience in. Finally, end user recruitment can be expensive, therefore Phase 2 acts as a way to remove many of the usability problems, however simple or complex they may be, before the prototype reaches end users. Experts may also be expensive to hire or recruit; however, within a research group or enterprise, usability inspection groups can be formed from stakeholders, designers, and developers who may already be involved in a project or related projects. Those not experienced in usability can be trained in how to analyze prototypes using heuristics.

One of the requirements of the methodology was to create an emphasis on rapid prototyping and evaluation, which is made possible in the methodology by introducing paper prototyping activities in Phase 1 and so-called discount usability engineering techniques in Phase 2. This emphasis on rapidity may lead to depreciation in quality. However, our methodology emphasizes the need for documentation and review after each phase. This will ensure that changes that have been recommended are disseminated, prioritized, and implemented before the next phase begins [71]. Ultimately, the quality and design of the testing and evaluations will dictate the quality and efficiency of the user feedback and what changes need to be made; this is why having a dedicated usability engineer on a design team is important [72].

In terms of measurability, how do we know our methodology has provided any improvement or is measurably better than other methodologies? This is hard to measure and would only be realistic if we applied different methodologies to the design of the same product. In this paper, we have identified many different methodologies that have been applied to the design and development of connected health and other similar medical devices. However, we identified a lack of standardized and guided approaches. Therefore, we wanted to derive a methodology that was guided by the principles and steps described in ISO 9241-210 and that has explicitly described steps and activities that other designers and engineers can follow. If this methodology is used in the future and is adopted by others, then we can start to measure its true effect and measure what its shortcomings may be, leading to improved HCD methodologies in the future. The application of the methodology to the WIISEL system and the subsequent results of this application will be explored in more detail in a separate paper.

### Conclusions

We conclude that our methodology brings a simple yet robust structure to HCD and development, while maintaining a rapid approach that will suit modern design and usability engineering teams in fast-paced and competitive industries. We have described in detail the activities that can be carried out in each phase. We have also presented our justification for this methodology and why we consider it to be a flexible and useful methodology, particularly for improving the usability, human factors, and user experience of devices and systems to be used for medical purposes.

## Abbreviations

ASQ: After-Scenario Questionnaire
CACP: Center for Advanced Communications Policy
CÚRAM: Centre for Research in Medical Devices
FDA: Food and Drug Administration
FP7: Seventh Framework Programme for Research
GP: general practitioner
HCD: human-centered design
IEC: International Electrotechnical Commission
ISO: International Organization for Standardization
SUS: System Usability Scale
UCD: user-centered design
UI: user interface
TLX: Task Load Index
WIISEL: Wireless Insole for Independent and Safe Elderly Living
